# Clinical Characteristics of Patients with Isolated Calf Vein Thrombosis in a Large Teaching Hospital

**DOI:** 10.1155/2011/414093

**Published:** 2011-04-07

**Authors:** Santin Brian, B. Fries Richard, Satiani Bhagwan

**Affiliations:** ^1^Mount Carmel Medical Center, Columbus, OH 43213, USA; ^2^Division of Vascular Diseases & Surgery, The Ohio State University Heart and Vascular Center and the Vascular Laboratory, The Ohio State University Medical Center, Columbus, OH 43210, USA

## Abstract

*Objective*. To identify the clinical characteristics of a patient population newly diagnosed with acute isolated calf deep venous thrombosis (ICDVT) by duplex ultrasound scan (DUS). *Methods*. A retrospective review of the records of 100 consecutive patients diagnosed with ICDVT by DUS was conducted. *Results*. Patients (59% male) were predominantly Caucasian (86%) and inpatients (69%) with an average age of 53 years. The most frequent risk factors were malignancy (22%), immobility (18%), and previous DVT (13%). Thrombus was present in named tibial veins in 58% and muscular branches in 42%. The peroneal vein was most frequently involved (39/117, 33%) followed by the gastrocnemius veins (29/117, 22%) and muscular calf tributaries (14%). *Conclusions*. Our patient population with ICDVT was predominantly symptomatic, in-patient cohort with a high incidence of risk factors such as malignancy, immobility, previous DVT, trauma, and postoperative status. Partial or complete resolution was documented by DUS in 53%.

## 1. Introduction

Deep venous thrombosis (DVT) of the lower limb poses a significant threat to patients' lives each year with more than 100,000 cases reported annually and is related to over 200,000 hospitalizations each year in the United States [1]. Although proximal DVT is associated with a much greater incidence of pulmonary embolism, the entirely benign nature of isolated calf deep vein thrombosis (ICDVT) as summarized by Philbrick and Becker in 1988 has been largely disproved [2]. 

Although the natural history of ICDVT, the propagation rate, and incidence of PE have been documented, the patient population in these reports has been extremely varied and documented prior to aggressive anticoagulation. We therefore sought to define the clinical characteristics of a predominantly inpatient population with newly diagnosed ICDVT by duplex ultrasound performed in a large teaching hospital.

## 2. Methods

One hundred consecutive patients with newly diagnosed acute ICDVT by DUS in the Vascular Laboratory at The Ohio State University Medical Center beginning in July 1st 2006 were part of this retrospective study. We excluded five patients with incomplete records leaving 95 patients with 119 ICDVTs. All patients with a history of prior ICDVT were also excluded. Demographic information including risk factors, treatment, and followup was obtained from Vascular Laboratory records and the Information Warehouse. Data were analyzed using Microsoft Excel (Microsoft Corp, Redmond, Wash). 

Duplex scanning was performed by registered vascular technologists in an ICAVL accredited laboratory. The ultrasound machines used were a General Electric Logiq 9 (GE Healthcare Technologies: Waukesha, Wisconsin, USA), Philips iU 22s (Philips Healthcare: Bothell, WA USA), and a Zonare Zone system (Zonare Inc: Mountain View, CA). Scanning technique is performed in a standard fashion with compression using grey-scale transverse scanning and color imaging utilizing both transverse and longitudinal planes. Calf veins are imaged as above from the knee to the ankle in both transverse and longitudinal planes. The location of the thrombus but not the length was noted.

This study was approved by the Institutional Review Board at the Ohio State University Medical Center.

## 3. Results

During the eight-month study period, there were 4,276 lower extremity venous DUSs performed on 7,126 limbs. Of these exams, 875 scans resulted in a diagnosis of acute or chronic DVT including the 100 patients diagnosed with ICDVT. The average age of the patient population was 53 years. Just over half of the patients were male (59%; 56/95) and 86% (82/95) were Caucasian. Approximately 69% (66/95) of the ICDVTs were diagnosed during an inpatient admission. 

The most common indication for requesting the DUS was edema (28%) (Table 1). Eleven patients had a recent diagnosis of a pulmonary embolus which led to the duplex examination. 

Risk factors and comorbidities are shown in Table 2. In the study population, diabetes mellitus (25%) and acute or chronic renal failure (18%) were the most common comorbidities. As would be expected in a large inpatient population, malignancy (20%), immobility (18%), and previous proximal DVT (13%) were frequent risk factors.

Table 3 outlines the location of the ICDVTs diagnosed by DUS. Both lower extremities were affected in nine percent. The most commonly involved calf vein was the peroneal vein (32%) followed by the gastrocnemius vein (22%). Named tibial veins were involved in 58% and muscular branches in 42% (Table 3). 

While long-term followup and treatment were not the focus of this study, at least one followup DUS venous exam was completed in 41 of the 95 patients (43%) (Table 4). Eighteen of the 41 patients (44%) demonstrated no change in the ICDVT. Lysis was seen in 53% with 16 (39%) showing partial recanalization of the affected vein, and 7 (17%) had evidence of complete resolution of the thrombus at a mean followup interval of 39 days (range 1, 180). Nineteen (20%) patients underwent a second followup duplex exam at an average of 107 days from their original diagnosis (range 4, 631). Equal numbers of patients demonstrated no change in the DVT and complete recanalization (37%). A third followup ultrasound exam was completed on 8 patients, all but one of these failed to reveal any change in the thrombus. 

No progression was noted in any patient that had a followup DUS.

## 4. Discussion

We report on a large series of patients with newly diagnosed ICDVT and identify and compare the principal characteristics of this patient population to other similar reports [3–8]. The majority of patients, identified in this study through a query of our vascular laboratory database, were inpatient with an average age of 53 years. Since 69% of patients in our cohort were inpatients, the risk factors were expectedly malignancy (19%), immobilization (18%), and postoperative or trauma patients (14%). This is similar to other reports in the literature [9]. 

The prevalence of ICDVT depends on whether the population being studied is asymptomatic and being screened or symptomatic. In symptomatic patients, the prevalence of ICDVT is reported as being 5–12%, and for high risk groups undergoing screening following joint replacement, for instance, it is 15% or higher [10, 11]. As pointed out, almost two-thirds of patients in this series were inpatients (and all symptomatic) even though the inpatient to outpatient ratio in our laboratory is approximately equal. In the same time frame as our study, the incidence of a positive DUS study (acute or chronic DVT) was 20% (875/4276), and the incidence of ICDVT was 2.3% (100/4276). ICDVT represented 11.4% of all positive DUS studies in the same time period.

The usual DUS of the calf performed to detect DVT consists of scanning the paired posterior tibial, peroneal, and anterior tibial veins. In addition, the nonpaired muscular veins (gastrocnemial and soleal) are also imaged. Most studies, like ours, report the peroneal vein as the most common site of ICDVT although some have reported that the site and size of thrombosis do not differ significantly amongst the calf veins [4]. The rarity of involvement of the anterior tibial vein in patients with ICDVT is confirmed in our study. Masuda et al. noted that of 58 limbs in 54 patients, 12% had gastrocnemius vein involvement and 0% anterior tibial vein involvement, similar to our results [3]. For this reason, some vascular laboratories may not even scan the anterior tibial vein in patients referred for a lower extremity DUS. Mattos et al. reviewed 655 limbs with DVT and concluded that, while calf veins should always be assessed when performing a lower extremity duplex exam, the anterior tibial veins may not necessarily be visualized as the incidence of thrombosis is negligible [7]. The incidence of muscular vein thrombosis in the calf varies from 12.5 to 15% [5].

The immediate and late consequences of ICDVT have been debated for many years. Propagation into the proximal larger veins and pulmonary embolism is of great clinical relevance because these events require aggressive anticoagulation even by clinicians who prefer not to treat patients with ICDVT. Labropoulos et al. found propagation of a calf clot in 27% and an incidence of pulmonary embolus in 2% in a series of 52 limbs with ICDVT [4]. A series by Gillet et al. reported a 7% incidence of pulmonary embolus from ICDVT [5]. Still others have reported similar findings of proximal propagation rates of ICDVTs [12]. Despite treatment with anticoagulants, there are reports of propagation of the thrombus in patients with ICDVT. Labropoulos et al. reported a 13% propagation rate of ICDVTs to the proximal venous system [4]. However, Parisi and colleagues showed evidence of propagation into the popliteal or thigh veins of only 2.9% of ICDVTs [13]. Interestingly, in Kazmers et al.'s review of over 3000 patients, those who were found to have propagation of a calf DVT were more likely to have cancer than not (35% versus 7.8%) [9]. We did not detect any propagation in the 41 patients with ICDVT in our series with the first followup DUS performed at an average of 39 days. In addition, although this was not a prospective series nor were patients subjected to routine ventilation/perfusion scans, no pulmonary embolic episodes were recorded in the medical record.

Besides proximal propagation and pulmonary embolism, long-term chronic reflux has also been documented after ICDVT. A 1998 study reported that 4% of patients experienced propagation of the calf vein thrombosis into the more proximal popliteal or thigh veins and 30% of patients developed venous reflux disease [3]. Meissner et al. corroborated these findings by demonstrating a 24% incidence of venous reflux disease at one-year followup in their study population with ICDVTs [6]. Our followup by DUS was not long enough to comment on late chronic venous disease.

*Limitations*. Aside from the retrospective nature of our study, the lack of followup beyond what was available in hospital records and on repeat ultrasound reports restricts us from reporting on the late natural history of ICDVT. In addition, we do not have data on the duration or the type of anticoagulant therapy, making it impossible to report on a correlation between the resolution of the thrombus and anticoagulant therapy. 

Spontaneous lysis of thrombus generally occurs within the first six weeks following diagnosis of DVT [14]. Masuda and Meissner report lysis in patients with ICDVT in over 50% of patients within three months. Although our followup interval was relatively short, 54% either resolved completely (17%) or partially (39%) whereas 44% demonstrated no change. The critical dilemma for clinicians is the inability to tailor the duration and intensity of anticoagulation to the probability and speed of thrombus resolution. As Labropoulos and colleagues point out, thrombus remodeling does seem to be correlated with the size, location, and pattern of thrombosis [4]. Techniques that measure the actual thrombus load, rather than the location or extent of thrombus, may provide more objective information on which to base clinical decisions upon [15].

## Figures and Tables

**Table 1:** Indications for duplex scan.

	*N*	Percent
Pain/Edema	20	21
Pain	21	22
Edema	27	28
Respiratory distress	13	14
Suspected pulmonary embolus	11	12
Screening after immobility	2	2
Acute stroke	1	1

**Table 2:** Comorbidities and risk factors.

Comorbidities	*N*	Percent
Cancer	19	20
Immobility	17	18
Previous thigh DVT	12	13
Postoperative/Trauma	14	14
Hypercoagulable state	2	2
Hypertension	11	12
Patent foramen ovale	1	1
Pregnancy	1	1
Acute CVA	1	1
Varicosities	1	1
Diabetes mellitus	24	25
Lymphoma	2	2
Renal failure	16	17
Chronic	7	7
Acute	9	9

**Table 3:** Location of calf DVTs.

Location	*N*	Percent
Right	66	55
Left	42	35
Bilateral	11	9
Muscular calf vein	16	13
Anterior tibial vein	0	0
Posterior tibial vein	17	14
Peroneal vein	39	32
Gastrocnemius vein	26	22
Soleal vein	7	6
Tibial trunk	12	10

**Table 4:** Followup.

	*N *	Percent
First followup	41	43
Interval		
Mean	39	
Median	13	
Mode	7	
Range	1, 180	

Second followup	19	20
Interval		
Mean	107	
Median	51	
Mode	n/a	
Range	4, 631	

Third followup	8	8
Interval		
Mean	125	
Median	130	
Mode	n/a	
Range	7, 227	

Fourth followup	1	1
Interval	14	
